# Assessment of serology and spirometry and the combination of both to complement microbiological isolation for earlier detection of *Pseudomonas aeruginosa* infection in children with cystic fibrosis

**DOI:** 10.1186/s12890-016-0327-9

**Published:** 2016-11-25

**Authors:** Ana Kotnik Pirš, Uroš Krivec, Saša Simčič, Katja Seme

**Affiliations:** 1 Department of Pediatrics, Unit for Pulmonary Diseases, University Children’s Hospital, University Medical Center Ljubljana, Bohoričeva 20, SI 1000 Ljubljana, Slovenia; 2 Department of Pediatrics, Faculty of Medicine, University of Ljubljana, Bohoričeva 20, 1000 Ljubljana, Slovenia; 3 Laboratory for Humoral Immunology, Institute of Microbiology and Immunology, Faculty of Medicine, University of Ljubljana, Zaloška 4, 1000 Ljubljana, Slovenia; 4 Laboratory for Diagnostics of Respiratory Infections, Institute of Microbiology and Immunology, Faculty of Medicine, University of Ljubljana, Zaloška 4, 1000 Ljubljana, Slovenia

**Keywords:** Early *Pseudomonas aeruginosa* infection detection, Cystic fibrosis, Children, Microbiological isolation

## Abstract

**Background:**

The aim of this study was to assess whether serology and spirometry and the combination of both can complement culture-based detection for earlier recognition of *Pseudomonas aeruginosa* infection in children with cystic fibrosis.

**Methods:**

A 4 year longitudinal prospective study that included 67 Slovenian children with cystic fibrosis with a mean age of 10.5 years was conducted. Serology, spirometry and a scoring system combining serology and spirometry were assessed and compared. Infection was confirmed with isolation of *Pseudomonas aeruginosa* from respiratory samples.

**Results:**

There was a significantly positive correlation between serology and the combination of serology and spirometry and *Pseudomonas aeruginosa* isolation (*P* < 0.01 for both) and a significantly negative correlation between spirometry and *Pseudomonas aeruginosa* isolation (*P* < 0.05). An increase in serology for 1 ELISA unit increased the possibility of *Pseudomonas aeruginosa* isolation 1.6 times. A fall in FEV1% predicted for 10% increased the possibility of *Pseudomonas aeruginosa* isolation 9.8 times. Binary logistic regression analysis was used to determine the odds ratios and 95% confidence intervals for all three approaches. Serology had the highest specificity (0.80) and the combination of serology and spirometry the highest sensitivity (0.90). Both had a high negative predictive value (0.93 and 0.79 respectively).

**Conclusion:**

Using serology and the combination of serology and lung function measurement can be beneficial for earlier detection of infection with *Pseudomonas aeruginosa* in children with cystic fibrosis when done simultaneously with standard culture-based detection from respiratory samples.

## Background

Chronic infection with *Pseudomonas aeruginosa* (*P. aeruginosa*) is a known cause of increased morbidity and mortality in patients with cystic fibrosis (CF) [1–4]. Early detection of *P. aeruginosa* infection enables early antibiotic therapy and enhances the chances of successful eradication. Detection of every new infection with *P. aeruginosa* is important – in never infected patients the first infection, in intermittently infected a new infection after successful eradication and in chronically infected an infection with a new non-mucoid strain of *P. aeruginosa* [2, 5–7]. When *P. aeruginosa* remains in the lungs of CF patients for longer periods, the strains change to mucoid type, which makes eradication practically impossible [1, 2, 8]. To evaluate the presence of *P. aeruginosa* in the lower airways, culture-based detection is preferably carried out from samples that reflect microbiota mostly from the lower airways such as sputum or bronchoalveolar lavage (BAL) [9–11]. Sputum is usually produced in patients with progressive CF or those that are in exacerbation and acquisition of BAL in children requires bronchoscopy under general anesthesia. In non-sputum-producing CF patients induced sputum has been shown to improve detection of pathogens, including *P. aeruginosa* [12]. Induced sputum can be difficult to acquire in children under the age of 5 years and in such instance can be replaced by deep throat aspirates or swabs [13]. Although deep throat swabs and aspirates are convenient to acquire in small children, they can reflect microbiota also from the upper airways and are an approximation of the true microbiological state of the lower airways [14]. Indirect detection methods such as determination of anti-*P. aeruginosa* antibodies in serum of patients (serology) have been shown to be useful for confirmation of chronically infected patients, who usually have very high antibody values. In intermittently infected patients interpretation of serology results can sometimes be difficult [15–20]. In some patients antibody values can be above the cut-off value for a positive test even when *P. aeruginosa* is not isolated in respiratory samples. If such patients have signs of an exacerbation or a progressive worsening of their clinical status and lung function parameters, further clinical investigations are due as it is possible that *P. aeruginosa* is not isolated because of technical difficulties even though it is present in the lower airways. On such occasions new methods that would be non-invasive and could increase the possibility of earlier detection of infection in children and non-sputum-producing CF patients would be beneficial.

The aim of our study was to assess and compare three different approaches for earlier detection of *P. aeruginosa* infection in children and adolescents with cystic fibrosis. Used complimentarily with isolation from respiratory samples, serology, lung function testing and a scoring system combining serology and spirometry were evaluated and compared. Infection was confirmed with isolation of *P. aeruginosa* from respiratory samples.

## Methods

### Study design and participants

There are currently 75 patients managed at the Center for Children and Adolescents with CF at the University Children’s Hospital in Ljubljana, Slovenia. Here, the current standards of care [21, 22], standards for diagnosis [23, 24] and guidelines for management and treatment of lung infection [25] in CF patients are followed.

This study included 67 patients with an established diagnosis of CF, who attended the Center between 2011 and 2015. A diagnosis of CF was made according to the current published guidelines and was confirmed if a patient had two positive sweat tests on two occasions and two disease causing mutations of the *CFTR* gene determined [23, 24].

**Fig. 1:**
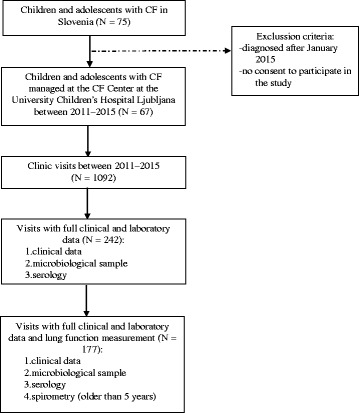
Study design of the longitudinal prospective study of children and adolescents with CF managed at the Center for Children and Adolescents with Cystic Fibrosis at the University Children’s Hospital in Ljubljana, Slovenia

Ethics approval for the study was granted by the Slovenian National Ethics Committee and written consent from the patient, parents, or caregivers was acquired before enrollment. The patients were seen at regular 3-monthly out-patient visits, yearly check-ups and at exacerbations. At all visits the clinical status and spirometry (in patients older than 5 years or younger, if able to perform the test) were evaluated and respiratory samples for microbiological isolation were obtained. Serum for measuring *P. aeruginosa* antibodies was collected simultaneously with clinical measurements and microbiological sampling at yearly check-ups and at exacerbations. In all patients at least two consequent serum samples for the detection of anti-*P. aeruginosa* antibodies were collected, one at the inclusion and the other at the end of the study. On the mean 3.8 (SD ± 1.2) serum samples were collected per patient during the study. At the end of the study the patients were clustered into three groups regarding the modified Leeds criteria: never infected (when *P. aeruginosa* had never been isolated), intermittently infected (when *P. aeruginosa* had been isolated in less than 50% of samples taken in 1 year) and chronically infected (when *P. aeruginosa* had been isolated in more than 50% of samples taken in 1 year) [26]. The design of the study is presented in Fig. 1.

### Materials

Respiratory samples for microbiological isolation were obtained based on the patient’s ability to expectorate. Sputum was the preferred sample. In young children and patients unable to expectorate, induced sputum was acquired. In very young children and babies, deep throat aspirates were acquired. If a sample could not be acquired in any of the above ways and infection with *P. aeruginosa* was probable, BAL was taken during bronchoscopy.

In the Center for Children and Adolescents with CF in Ljubljana, deep throat aspirates instead of swabs are collected in children unable to produce sputum or induced sputum. The technique of taking a deep throat aspirate is similar to the second part of acquiring an induced sputum sample in small children [13]. The difference is that no inhalation of hypertonic saline is done beforehand. A sterile catheter is used. The suction catheter is inserted into the pharynx. To minimize contamination of the specimen with secretions from the oropharynx, suction is applied to the catheter only after it has been inserted into the pharynx and is discontinued before it is withdrawn.

Respiratory samples were processed according to the current valid guidelines [27]. Briefly, sputum samples were initially homogenized by using dithiothreitol (Sputasol, Oxoid, Basingstoke, UK). Samples were inoculated on 5% sheep’s blood agar, chocolate agar, MacConkey agar, chromogenic agar for the detection of *Staphylococcus aureus*, *Burkholderia cepacia* selective agar (BCC), Sabouraud dextrose agar and incubated at 35 ± 1 °C for 48 h, with the exception of Sabouraud agar and BCC which were incubated for 7 and 5 days, respectively. Plates were examined daily. MALDI-TOF mass spectrometry using Biotyper Microflex LT and MALDI Biotyper 3.0 software (Bruker Daltonik, Bremen, Germany) was used for identification of isolates.

Lung function was measured with standard spirometry in patients over 5 years of age and in younger patients who were able to perform the test. The forced expiratory volume in one second percent predicted value for age, gender, and height (FEV1%) was used as a determinant of lung function, based on Zapletal spirometry pediatric reference values [28].

A Pseudomonas-CF-IgG test (Statens serum Institut, Denmark, Art. No. 64742) was used for the quantitative measurement of IgG antibody values of *P. aeruginosa* in human serum samples. The antigens used to coat an enzyme-linked immunoassay (ELISA) plate were a mixture of antigens from *P. aeruginosa* serotypes O-1 to O-17, which are the most commonly isolated serotypes in respiratory samples of CF patients [15, 16]. All serum samples of CF patients were tested in duplicates. As required by the manufacturer, a standard curve in a range of 0 to 50 ELISA units with standard pooled human antiserum diluted to 1:2.000 defined as 50 ELISA units was used to measure the *P. aeruginosa* IgG concentration in CF patient samples.

### Statistical analysis

IBM SPSS version 22 was used for statistical analysis. Pearson’s correlation and the chi-square test were used to calculate the correlation between results of serology, spirometry and the combination of serology and spirometry and the results of *P. aeruginosa* isolation. Receiver operator characteristics curves (ROC curves) were constructed for all three approaches and cut-off values determined. The area under the curve (AUC), sensitivity, specificity, positive predictive value (PPV), and negative predictive value (NPV) were calculated. Binary logistic regression analysis was used to calculate the odds ratio (OR), 95% confidence intervals (CI) and *P* values.

## Results

The study group included 67 patients with a confirmed diagnosis of CF. 29 (43%) patients were female and 38 (57%) male. Their mean age was 10.5 years (SD ± 5.9, range 0.3–23.1 years). At the end of the study they were clustered into three groups regarding the modified Leeds criteria. There were no statistically significant differences according to the number of participants, their gender and their mean age between groups of never and intermittently infected patients (*N* = 29 vs. *N* = 32; male:female ratio 17:12 vs. 16:16; age: M = 8.93 vs. 10.63 years). There were less participants in the chronically infected group and their mean age was higher (*N* = 6; M = 19.59 years; male:female ratio 2:4).

**Fig. 2:**
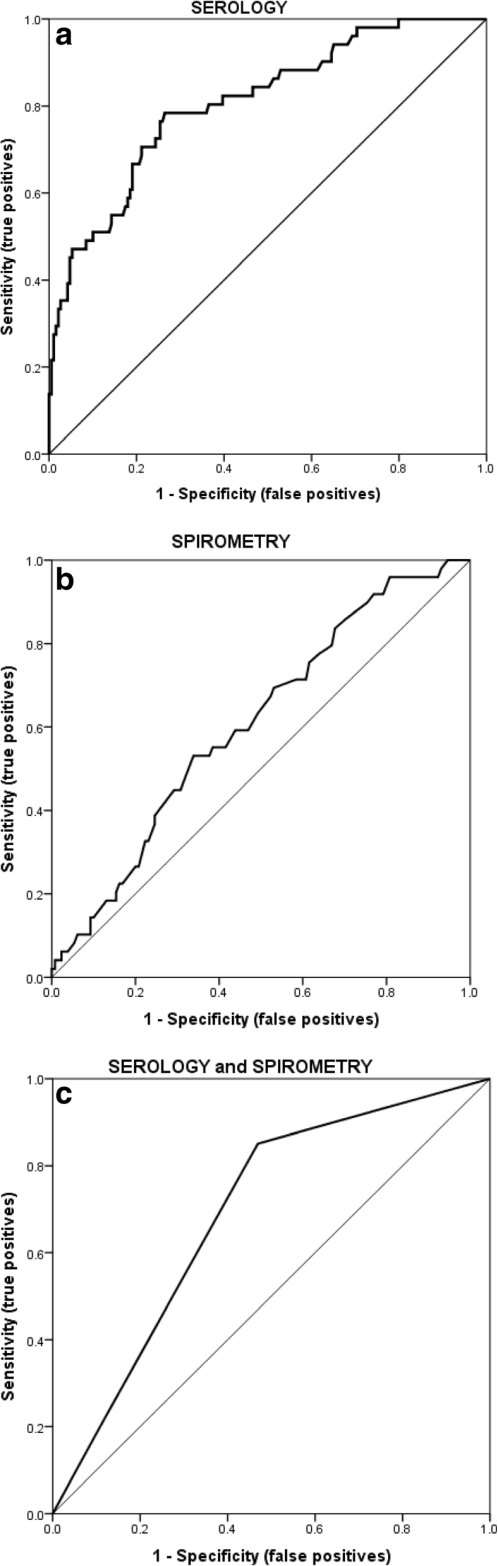
**a** ROC curve for serology. The cut-off point was specified from the ROC curve using the optimal intersection of specificity and sensitivity. Based on the drawn ROC curve, the cut-off point for serology was at 2.96 ELISA units. **b** ROC curve for spirometry. The cut-off point was specified from the ROC curve using the optimal intersection of specificity and sensitivity. Based on the drawn ROC curve, the cut-off point for spirometry was at 70% of FEV1 predicted for gender, age and height. **c** ROC curve for the combination of serology and spirometry. The cut-off point was specified from the ROC curve using the optimal intersection of specificity and sensitivity. Based on the drawn ROC curve, the cut-off point for the combination of serology and spirometry was at 2.96 ELISA units and an FEV1 at 70% of the predicted value for gender, age and height

A total of 1092 clinic visits were conducted during the 4 years of the study. The data from the visits was considered complete if it included all the clinical parameters, the result of isolation of *P. aeruginosa* from the respiratory sample and the serology result and in patients old enough to perform spirometry, the value of FEV1% predicted. Data from 242 visits was eligible for further study. At 177 of the 242 visits lung function could also be measured. From the 242 respiratory samples *P. aeruginosa* was isolated in 52 (21.5%) samples. To evaluate the accuracy of the used diagnostic approaches to predict *P. aeruginosa* infection, ROC curves were constructed for serology, spirometry and the combination of serology and spirometry (Fig. 2a, b and c).

Cut-off values were specified from the ROC curves using the optimal intersection of specificity and sensitivity. Based on the data, the cut-off point for serology was at 2.96 ELISA units, which was the same as recommended by the manufacturer of the test. Based on the ROC curve for spirometry the FEV1% cut-off value was at 70%. When analyzing results of the combination of serology and spirometry three different outcomes were possible: normal serology and spirometry (ELISA < 2.96 EU and FEV1 > 70%), abnormal serology or spirometry (ELISA > 2.96 EU or FEV1 < 70%), or abnormal serology and spirometry (ELISA > 2.96 EU and FEV1 < 70%). When serology and spirometry were normal the result was marked as 0, when either serology or spirometry were abnormal the result was marked as 1, and when both were abnormal the result was marked as 2. The possible outcomes and scores and their correlation with *P. aeruginosa* isolation from respiratory samples are presented in Table 1.

**Table 1:** Correlation between the scoring system using the combination of serology and spirometry and isolation of *P. aeruginosa* in the patients enrolled in the study

Possible outcome for the combination of serology and spirometry	Score	Isolation of *P. aeruginosa*		Number of visits
		NO	YES	
ELISA < 2.96 EU and FEV1 > 70%	0	70	7	*N* = 77
ELISA > 2.96 EU or FEV1 < 70%	1	45	30	*N* = 75
ELISA > 2.96 EU and FEV1 < 70%	2	10	15	*N* = 25
Number of samples		*N* = 125	*N* = 52	*N* = 177

The AUC of the ROC curves, sensitivity, specificity, PPV and NPV of the in the study used diagnostic approaches to predict *P. aeruginosa* infection were calculated. Serology had the highest AUC (0.81), sensitivity (0.80) and NPV (0.93). It was followed by the combination of serology and spirometry with an AUC of 0.70 and the highest specificity of all of the three used methods (0.90). The NPV was also high (0.79). Spirometry alone had the lowest AUC (0.61), but high specificity (0.83) and a NPV of 0.71. The data for all three used approaches are presented in Table 2.

**Table 2:** Comparison of the area under the curve, sensitivity, specificity, positive and negative predictive values between serology, spirometry and the combination of serology and spirometry for prediction of *P. aeruginosa* infection

Diagnostic test	AUC	Sensitivity	Specificity	PPV	NPV	Number of visits
Serology	0.81 (SE 0.03, *P* < 0.02, CI 0.74–0.88)	0.80	0.67	0.39	0.93	N = 242
Spirometry	0.61 (SE 0.05, *P* < 0.05, CI 0.52–0.70)	0.29	0.83	0.43	0.71	*N* = 177
Serology and spirometry	0.70 (SE 0.04, *P* < 0.02, CI 0.61–0.78	0.28	0.90	0.46	0.79	*N* = 177

*Legend*: *AUC* Area under the curve, *SE* Standard error, CI – 95% confidence interval, *NPV* Negative predictive value, *PPV* Positive predictive value, *N* Number of visits

Pearson’s correlation was used to define the correlation between serology and spirometry and isolation of *P. aeruginosa* from the collected respiratory samples. To evaluate the correlation between the combination of serology and spirometry and isolation of *P. aeruginosa* the chi-square test was used*.* A significantly positive correlation between serology and the combination of serology and spirometry and *P. aeruginosa* isolation was confirmed (*P* < 0.01 for both). There was a significantly negative correlation between spirometry and isolation of *P. aeruginosa* (*P* < 0.05). A higher value of serology or a higher score on the combined test and a lower value of FEV1% predicted correlated with a higher possibility of *P. aeruginosa* isolation.

Binary logistic regression analysis was performed to evaluate the predictive value of each of the used approaches for possible *P. aeruginosa* isolation. If the value of serology was over 2.96, there was a 83.3% chance that *P. aeruginosa* would be isolated (*P* < 0.01). If the FEV1% predicted value was below 70% there was a 72.6% possibility that *P. aeruginosa* would be isolated (*P* < 0.05). If the FEV1% predicted was under 70% or serology over 2.96 ELISA units there was a 73.4% chance of *P. aeruginosa* being isolated (*P* < 0.01). An increase of serology for 1 ELISA unit increased the possibility of *P. aeruginosa* isolation 1.6 times. A fall in FEV1 for 10% increased the possibility of *P. aeruginosa* isolation 9.8 times. An increase in the score of the combination of serology and spirometry increased the possibility of *P. aeruginosa* isolation 0.15 times. The results of binary logistic regression analysis of the used approaches associated with *P. aeruginosa* isolation are presented in Table 3.

**Table 3:** Binary logistic regression analysis of serology, spirometry and their combination with *P. aeruginosa* isolation

Approach	Odds ratio	95% Confidence interval	*P* value
Serology	1.60	1.40–1.85	<0.01
Spirometry	0.98	0.96–1.00	<0.05
Combination of serology and spirometry	0.15	0.05–0.46	<0.01

**Fig. 3:**
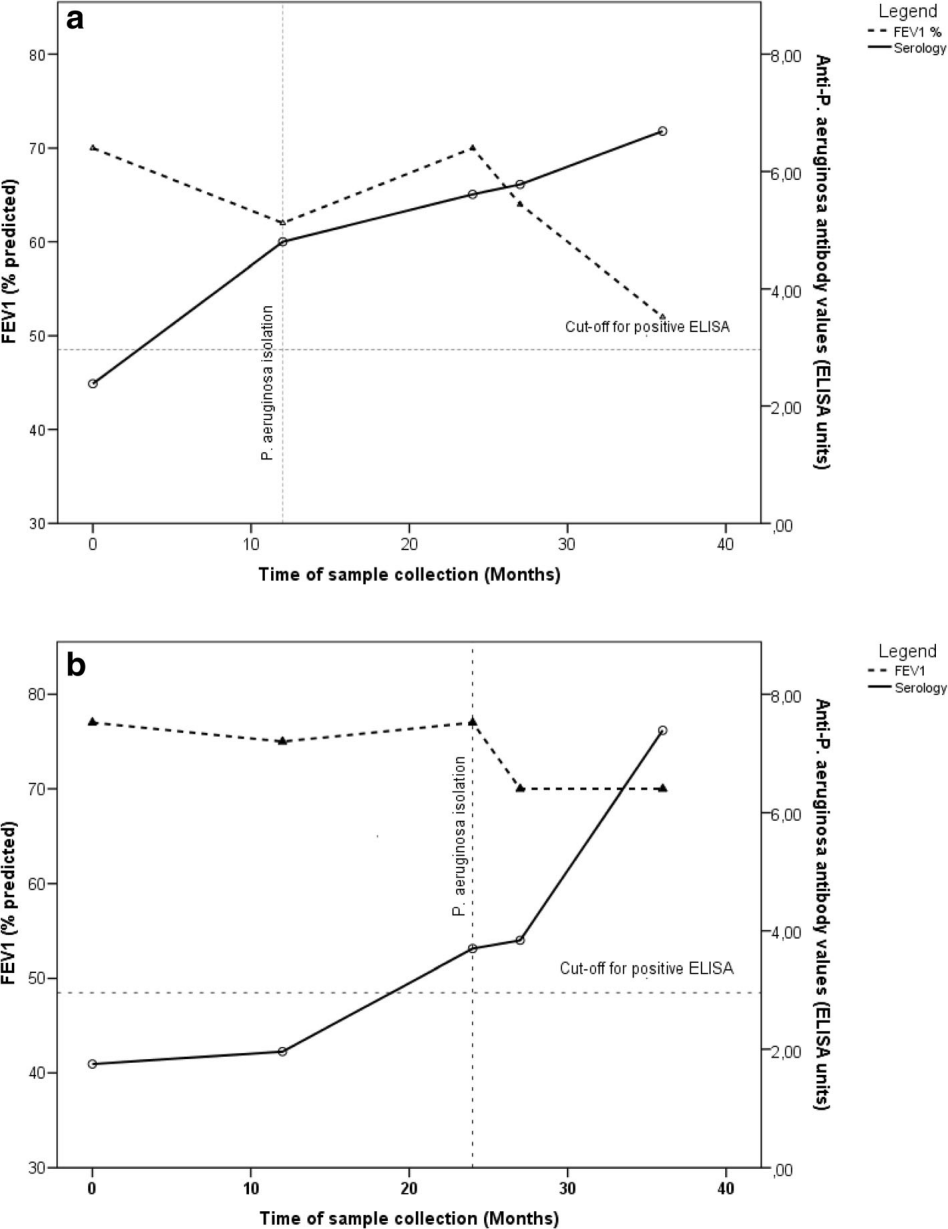
**a** and **b** Dynamics of lung function and anti-P*. aeruginosa* antibody values over time in two identical twins from the intermittently infected group. *P. aeruginosa* had been last isolated from their sputum 2 years before inclusion in the study. Eradication therapy had been completed and was considered successful at that time according to the guidelines [25]. Isolation of *P. aeruginosa*, aggravation of lung function and a rise in anti-*P. aeruginosa* antibodies was observed a year apart in the two twins. *P. aeruginosa* had been further intermittently isolated in both patients after the end of the study

The change in antibody values and a change in FEV1% predicted during the study were also evaluated. When assessing the changes of serology and lung function over time in individual patients, dynamics of both could be seen. The dynamics in serology and spirometry over time in association with *P. aeruginosa* isolation from two patients from the intermittently infected group is shown in Fig. 3. The two patients are identical twins, carrying the same two disease causing mutations of the *CFTR* gene. They live in the same household and had sputum samples acquired at the same time during the study. They were 13 years old at the inclusion into the study. *P. aeruginosa* had been isolated in their sputum 2 years before inclusion and had then been eradicated. In the first twin a fall in lung function and a rise in antibody values were determined 12 months after inclusion into the study. At the same time *P. aeruginosa* had been isolated and eradication therapy started. Despite eradication therapy, which was considered successful since *P. aeruginosa* had not been isolated in any of the samples taken after the end of eradication, an elevation of antibody values continued and a further decline in lung function followed especially after month 24 of the study (Fig. 3a). After the end of the study *P. aeruginosa* had been again intermittently isolated. In the second twin, a year later, a rise of anti-*P. aeruginosa* antibodies together with a fall in FEV1% predicted was observed. At the same time *P. aeruginosa* was isolated (Fig. 3b).

**Fig. 4:**
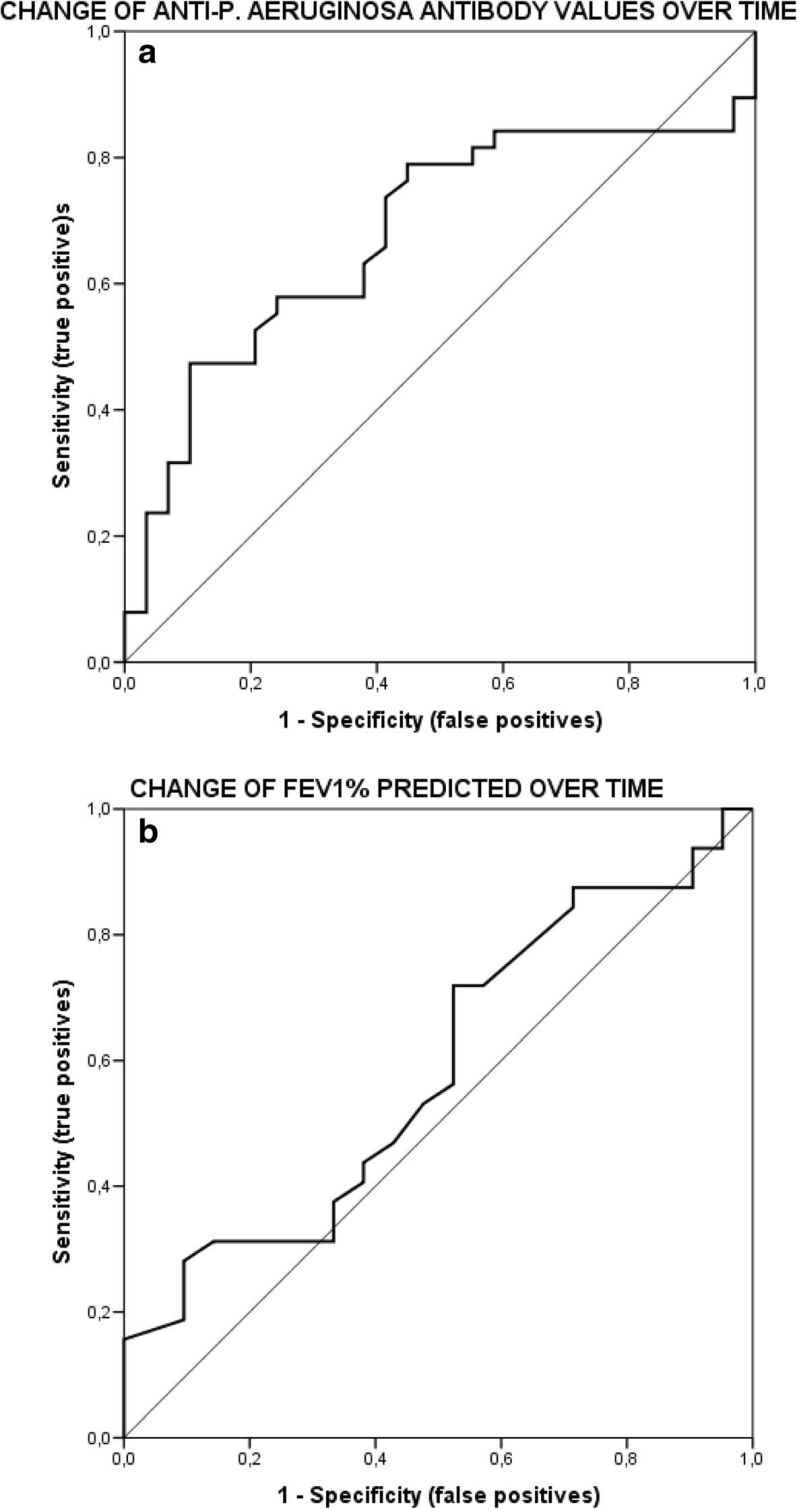
**a** ROC curve for the change in serology values over time. Based on the joined data of all participants, an optimal cut-off value for the change in serology over the time of the study could not be determined from the constructed ROC curve. The AUC for the change of serology over time was 0.63. **b** ROC curve for the change in spirometry over time. Based on the joined data of all participants, an optimal cut-off value for the change in spirometry over the time of the study could not be determined from the constructed ROC curve. The AUC for the change of spirometry over time was 0.58

When evaluating the data for all participants, a statistically significant correlation of the change in FEV1% predicted and the change in serology values over time could not be confirmed (*P* > 0.05 for both). ROC curves for the changes of serology and lung function over time were constructed. The AUC for the change of serology over time was 0.63, *P* < 0.02. For the change of FEV1% predicted over time the AUC was 0.58, *P* > 0.05. The ROC curves are presented in Fig. 4a, b.

## Discussion

Chronic infection with *P. aeruginosa* is a known risk factor for higher morbidity and shorter survival of patients with CF [1–4]. Early detection of infection can enable the start of early eradication antibiotic therapy, which can postpone or prevent chronic infection [5–7, 29, 30]. Culture-based detection of *P. aeruginosa* in respiratory samples is the current gold standard for infection confirmation [27]. In children acquisition of sputum is not always possible and induced sputum, deep throat swabs or aspirates are used as a surrogate [10–12, 14]. Non invasive methods that could complement isolation of *P. aeruginosa* for earlier detection of infection in children with CF would be beneficial.

According to this and the previously published studies on *P. aeruginosa* serology [15–19], values of anti-*P. aeruginosa* antibodies are important for evaluating the possibility of *P. aeruginosa* infection in patients with CF. In the presented study serology had high sensitivity (0.80) and a good AUC (0.81) to predict *P. aeruginosa* infection.

A scoring system using the combination of anti-*P. aeruginosa* serum antibody values and lung function measurement, which has not been previously described, was developed and assessed for the first time on a group of children and adolescents with CF. The scoring system had a high specificity of 0.90 and NPV of 0.79, thus enabling a firm recognition of truly *P. aeruginosa* negative patients. Complementing standard microbiological isolation with the combination of serology and lung function measurement could therefore be useful for ruling out infection with *P. aeruginosa* as a cause of clinical deterioration. On the other hand, detecting high values of anti-*P. aeruginosa* antibodies in a patient with a decline of lung function could be helpful for making further decisions on how to manage the patient. The clinician would be encouraged to proceed with more invasive methods for microbiological sampling such as BAL, to acquire an adequate sample for culture-based infection confirmation. The result of isolation would then further influence the decision of starting antibiotic therapy as this should be started only if *P. aeruginosa* is isolated in the appropriate respiratory sample.

**Fig. 5:**
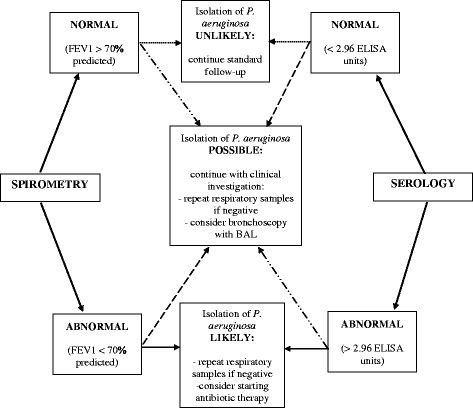
A proposed algorithm for the evaluation of the combination of serology and spirometry for assessing the possibility of *P. aeruginosa* infection. Results of serology and spirometry should always be interpreted together with the results of culture-based detection of *P. aeruginosa* in respiratory samples. The decision on starting antibiotic therapy should be based on the results of a positive culture and positive indirect methods should encourage the clinician to continue/repeat microbiological investigations. Legend:  normal serology and spirometry;  abnormal serology and normal spirometry;  normal serology and abnormal spirometry;  abnormal serology and spirometry

An algorithm that could be used on such occasions has been constructed and is presented in Fig. 5. In this study, the sensitivity and PPV of the scoring system to predict *P. aeruginosa* infection were low (0.28 and 0.46, respectively), which could cause positive patients to be missed if the test was used alone and only on rare occasions. The low sensitivity and PPV could be explained by the used cut-off value for spirometry, the mean age and the mean FEV1% predicted of our patients. An FEV1 below 70% was used as the cut-off value for predicting the possibility of infection with *P. aeruginosa* based on the calculated ROC curve for spirometry in this study. The mean age of the included patients was 10.5 years (SD ± 5.9, range 0.3–23.1 years) and the mean FEV1% predicted 84.9% (SD ± 18.57, range 30–124%). It is expected that younger patients have better lung function. This was also shown in our study. This could have been the cause for the low number of patients with an FEV1% under 70% which in turn caused a low number of truly positive patients when assessing spirometry and the combination of serology and spirometry for prediction of *P. aeruginosa* infection.

The youngest population of CF patients still remains challenging for diagnostics because they are not only unable to expectorate quality sputum or induced sputum samples but are also unable to perform spirometry. Other lung function testing methods such as the interrupter technique could be used in these patients but are not performed routinely because of technical difficulties and limitations assessing FEV1. The lung clearance index (LCI) as a lung function parameter derived from the multiple-breath washout test has been assessed in preschool children [31, 32] and could be useful in combination with serology and microbiological isolation to predict infection with *P. aeruginosa* in the future.

There are various ways to determine whether a patient is infected by specific microorganisms. All are based on identification of an organism in specific microbiological samples. Culture-based methods are currently the gold standard for the detection of *P. aeruginosa* in respiratory samples of CF patients [14, 27]. They are relatively inexpensive and standardized, and have the ability to identify only viable organisms, which can then be tested for antibiotic susceptibility and stored for further study [12]. On the other hand, culture-independent methods such as quantitative real-time polymerase chain reaction (PCR), 16S rRNA sequencing, and next-generation sequencing allow faster and precise identification of organisms if used with large sequence databases, but it is possible for non-viable organisms to also be detected [14, 33, 34]. Combination of serology with newer microbiological methods such as real time PCR for *P. aeruginosa* detection directly in respiratory samples has been studied in children with CF. In a study by da Silva et al. in 87 children with CF, three *P. aeruginosa* detection methods and their combinations were evaluated. *P. aeruginosa* was detected using culture in 48.2% of patients, PCR in 60.9% and serology in 43.6%. The difference between the used methods was not statistically significant, but PCR in combination with serology implied to be most useful for early *P. aeruginosa* detection [34]. Further studies combining optimized quantitative real-time PCR protocols with serology are needed to determine whether such an approach enables more efficient and timely *P. aeruginosa* detection.

In the presented study *P. aeruginosa* was in overall isolated from 21.5% of samples. It was isolated from 43% of samples taken at visits in which the value of anti-*P. aeruginosa* antibodies was over 2.96 ELISA units, from 20.9% of samples taken at visits in which the value of FEV1% predicted was under 70% predicted and from 14.7% of samples taken at visits in which either serology was over 2.96 ELISA units or FEV1% was under 70% predicted. If used without culture, serology would have overestimated the number of infections. In this study spirometry and the combination of serology and spirometry closely related the number of positive respiratory samples and estimated the number of infected patients well.

## Conclusions

The challenge of detecting infection with *P. aeruginosa* in the pediatric CF population as early as possible remains. Laboratory diagnostic methods are best interpreted together with the clinical state of the patient. It is hoped that combining new microbiological and physiological methods together with precise clinical observation on larger study groups will yield a good solution. It was the aim of the presented study to provide an approach that would enable the physician to be more vigilant. The results show that serology, spirometry and the combination of both can be a useful complementary approach for earlier *P. aeruginosa* infection detection in children with cystic fibrosis when used together with standard culture-based methods for infection confirmation. A change in serology, spirometry or both could alert the physician of a possible infection with *P. aeruginosa.* Consequently diagnostic procedures for direct detection/isolation of *P. aeruginosa* should be intensified, finally enabling an earlier start of targeted treatment in patients in whom the results of isolation would confirm infection.

## Abbreviations

AUC: Area under the curve
BAL: Bronchoalveolar lavage
CF: Cystic fibrosis
CI: 95% confidence interval
ELISA: Enzyme-linked immunoassay
FEV1%: Forced expiratory volume in one second percent predicted
LCI: Lung clearance index
MALDI-TOF MS: Matrix-assisted laser desorption/ionization time-of-flight mass spectrometry
NPV: Negative predictive value
OR: Odds ratio
*P. aeruginosa*: *Pseudomonas aeruginosa*
PCR: Polymerase chain reaction
PPV: Positive predictive value
ROC curve: Receiver operator characteristics curve
SD: Standard deviation
